# A Case Report of Typhoidal Acute Acalculous Cholecystitis

**DOI:** 10.1155/2014/171496

**Published:** 2014-06-26

**Authors:** Neeha Rajan, Imeldah Motoroko, Dilshan Udayasiri, Jo-Lyn McKenzie, Jason S. C. Tan, Adrian R. Tramontana

**Affiliations:** Department of General Surgery and Infectious Diseases, Western Footscray Hospital, Gordon Street, Footscray, Melbourne, VIC 3011, Australia

## Abstract

*Introduction*. Acalculous cholecystitis in the setting of typhoid fever in adults is an infrequent clinical encounter, reported sparsely in the literature. In this case report we review the presentation and management of enteric fever involving the biliary system and consider the literature surrounding this topic. The aim of this case report is to alert clinicians to the potential diagnosis of extraintestinal complications in the setting of typhoid fever in the returned traveller, requiring surgical intervention. *Presentation of Case*. We report the case of a 23-year-old woman with acalculous cholecystitis secondary to *Salmonella* Typhi. *Discussion*. There is scarce evidence surrounding the optimal treatment and prognosis of typhoidal acalculous cholecystitis. In the current case, surgical invention was favoured due to failure of medical management. *Conclusion*. Clinical judgement dictated surgical intervention in this case of typhoidal acute acalculous cholecystitis, and cholecystectomy was safely performed.

## 1. Introduction

Typhoid fever is a systemic bacteraemic infection caused by ingestion of* Salmonella enterica* serovar Typhi (*S.* Typhi) or* Salmonella* Paratyphi A (*S.* Paratyphi A). The infection is transmitted by the faecooral route. The incubation period is 5–30 days. As an acute illness, it typically presents with nonspecific symptoms of malaise, fatigue, fevers, abdominal pain, headache, and diarrhea [1].

Acute acalculous cholecystitis is an acute inflammation of the gallbladder in the absence of gallstones and accounts for 5% to 10% of all cases of acute cholecystitis [2]. Very rarely it is seen as a complication of typhoid fever. The hypothesis behind the emergence of this rare complication could be linked to the increased number of travellers and migrants from endemic areas and also could be as a result of the occurrence of multidrug resistant and more virulent forms of* Salmonella* infection [3]. There are numerous reports of paediatric acalculous cholecystitis related to typhoid infection in the developing world, but it remains a rare diagnosis in adults, especially in developed countries. We therefore present this complication of typhoid fever in Australia in a young woman.

## 2. Case

A 23-year-old woman presented to our hospital with a two-day history of fevers and vomiting, epigastric pain, malaise, and headache. Her past history included polycystic ovaries, gastritis with previous helicobacter eradication, and hypothyroidism. She had recently travelled to her country of birth, Pakistan, for eight weeks and had returned to Australia two days prior to admission. Two weeks after arriving in Pakistan, she developed nausea, vomiting, headache, anorexia, and myalgia. Abdominal ultrasound was normal and barium swallow demonstrated gastritis. She was managed with esomeprazole, zinc, and atorvastatin. On returning to Australia, in addition to ongoing nausea and headache, she developed fevers and epigastric pain. On presentation to our metropolitan hospital she was febrile with 39.9°C, pulse rate 96/min, blood pressure 108/72 mmHg, and respiratory rate 20/min. Physical exam revealed a soft abdomen with mild suprapubic tenderness. Rose spots were present on her anterior chest and no jaundice was noted. Her white cell count was normal, platelet count was mildly low, her C-reactive protein elevated, and she had mild transaminitis (see Table 1).

No abnormalities were detected on chest X-ray. The blood cultures grew* Salmonella* Typhi with resistance to ciprofloxacin and sensitivity to amoxicillin, ceftriaxone, and cotrimoxazole. Investigations for malaria, HIV, dengue, and viral hepatitis were all negative. Stool cultures were negative.

**Figure 1:**
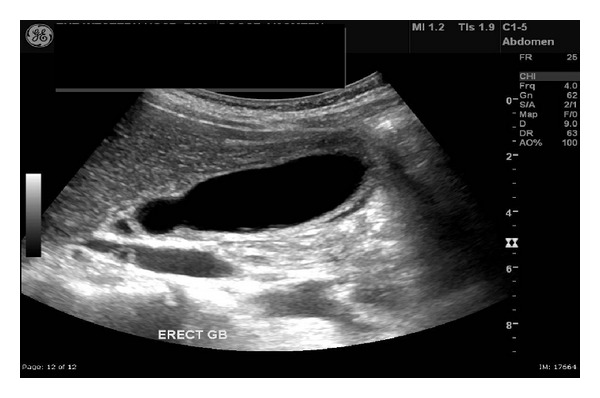
Gallbladder ultrasound showing a distended gallbladder, some pericholecystic fluid, and no gallstones.

She was treated with intravenous ceftriaxone 2 g daily. On the third day of her admission she developed severe right upper quadrant pain with localised peritonism despite her fever settling. Her LFTs were further deranged and abdominal ultrasound showed thickening of the gallbladder with pericholecystic fluid and probe tenderness, consistent with acalculous cholecystitis (see Figure 1).

She underwent laparoscopic cholecystectomy on the dame day. During the operation, a swollen, oedematous, and nonperforated gall bladder without stones was noted. The liver was noted to be normal, and there was a small amount of free fluid in the pelvis. Culture of the bile was not performed.

**Figure 2:**
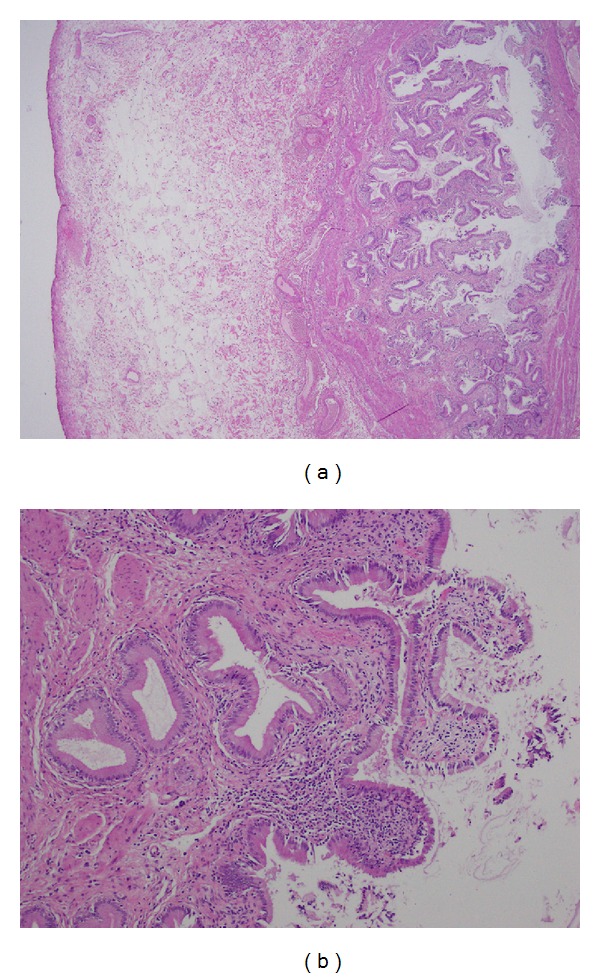
(a) Histology of gallbladder: low magnification (×4); (b) histology of gallbladder: high magnification showing chronic inflammatory cell infiltrate (×40).

The pathology of the resected gall bladder revealed chronic inflammatory cell infiltration and mild chronic cholecystitis (see Figures 2(a) and 2(b)).

She became febrile two days after the operation (temperature 38.8°C). Intravenous azithromycin was added to the antibiotic regime. A CT abdomen was unremarkable, and a chest X-ray revealed right lower lobe atelectasis. Intravenous meropenem was subsequently administered for the following two days. Her fever settled and she was discharged with oral azithromycin on day 10 of her admission.

## 3. Discussion

Acute hepatobiliary disease is an established but rare complication of* S.* Typhi and* S.* Paratyphi A infection [4]. Intestinal invasion into intestinal lymphoid tissue is followed by bacteraemia and macrophage phagocytosis of the bacteria. This produces a secondary bacteraemia which typically results in the acute phase of the disease. The bacteraemia establishes bacterial infection of the biliary system through the portal supply or retrograde biliary carriage [1]. This can lead to acute cholecystitis or long term colonisation of the gall bladder, particularly in those with gall stones. 2–5% of infected individuals develop a sustained infection of the gallbladder with shedding in the stool, producing a carrier state [4].

Acute acalculous cholecystitis is most commonly seen in critically ill patients suffering serious trauma, burns, severe sepsis, or long term total parenteral nutrition or postoperatively after major surgery [5]. Typhoid induced acalculous cholecystitis is primarily described in a paediatric population in endemic areas [6]. There have been few case reports in the adult literature describing the clinical characteristics of reported cases of acute acalculous cholecystitis associated with culture-proven typhoid fever in adults [2, 7–11] (see Table 2).

All these cases were acquired from endemic areas. Our patient had been to an endemic area (Pakistan) 8 weeks prior to admission. Other infections which have been described to cause acalculous cholecystitis are nontyphoidal Salmonellosis (*S. typhimurium* and* S. enteritidis*) in immunocompetent patients and cytomegalovirus and cryptosporidium in patients with advanced HIV [2].

The time of onset of typhoidal acute acalculous cholecystitis is variable and independent of any recognised attack of typhoid fever [7]. In some case reports, including this case, acute acalculous cholecystitis occurred in the first week [8, 11], and others have reported its occurrence in the second week [6, 12]. Furthermore its onset may be during convalescence or during a relapse [7].

In this case the pathological finding was discordant with the clinical presentation, given the fact that a lymphocytic infiltration of the gall bladder wall was more evident, indicating chronic inflammation, rather than a neutrophilic infiltration. The patient's duration of symptoms was less than 2 weeks and would not therefore be consistent with a clinical picture of chronic cholecystitis. It could be hypothesised that she had a primary infection during her travel and that* Salmonella* Typhi subsequently colonised and caused chronic inflammation of the gallbladder. Similar histopathological findings have been reported in an adult case of typhoidal acute acalculous cholecystitis [10].

Acute acalculous cholecystitis poses major diagnostic challenges. Physical exam and laboratory evaluation are unreliable [13]. Fever is generally present but other physical findings cannot be relied on, especially the physical examination of the abdomen [14]. Leukocytosis and jaundice are common but nonspecific in the setting of critical illness. Other biochemical assays of hepatic enzymes are of little help. The diagnosis of acute acalculous cholecystitis therefore often rests on radiological studies. Ultrasound of the gallbladder is the most accurate modality to diagnose acalculous cholecystitis [15]. Thickening of the gallbladder wall is the most reliable criterion with a specificity of 98.5% and sensitivity of 80% at 3.5 mm thickness [16–18]. Accordingly, a gallbladder wall thickness of 3.5 mm or greater is accepted to be diagnostic of acute acalculous cholecystitis. Other helpful sonographic findings include pericholecystic fluid or intramural gas, a sonolucent intramural layer or “halo” which represents intramural oedema [15]. CT seems to be accurate in the diagnosis of acute acalculous cholecystitis, based on a retrospective study comparing both modalities [19, 20]. The diagnostic criteria for acute acalculous cholecystitis by CT are similar to those described for sonography [21] (see Table 3).

Diagnosis is generally confirmed by isolation from blood cultures. Cultures of bone marrow aspirates have higher sensitivity but are rarely performed. Isolation from stool cultures can help establish the diagnosis in those with suggestive history but negative blood cultures [22].

Acute typhoidal acalculous cholecystitis appears to have a good prognosis with treatment as opposed to that which occurs in critical illness, which is associated with a high mortality and high rates of perforation and necrosis of the gall bladder [5]. In these cases, cholecystectomy is considered the optimal treatment for acute acalculous cholecystitis [5, 23]. Often critically ill patients are not well enough to undergo surgical treatment. In this setting percutaneous cholecystostomy is established as a safe and effective method of decompressing the gallbladder [24]. From the review of the reported paediatric of typhoidal acalculous cholecystitis cases, it appears that medical treatment without cholecystectomy may be appropriate [6, 12, 25–27]. Because of the limited number of cases in adults, the optimal treatment and prognosis of typhoidal acute acalculous cholecystitis have not been fully studied. Of the reported six cases, fifty percent have been treated successfully with medical therapy alone and 50% underwent cholecystectomy [2, 9–11]. The present patient underwent laparoscopic cholecystectomy based on the physical examination findings of local peritonism. Also, in this case the development of symptoms despite treatment with antibiotics constitutes an argument in favour of cholecystectomy as treatment.

## 4. Conclusion

As there is an emergence of multidrug resistant and more virulent forms of* Salmonella* infection, we may see more of the rarer complications of enteric fever such as acalculous cholecystitis. This case highlights typhoid as a cause of acalculous cholecystitis. This condition generally affects children in endemic areas but sometimes affects adults who have been to or returned from endemic areas. In the published literature, most cases have resolved with antibiotics alone. Cholecystectomy should be considered when there is no resolution of symptoms despite medical therapy, as was seen in this case. This case highlights the need to be vigilant and awareness of this infrequent complication and a high index of suspicion are required for effective treatment of this problem.

## Figures and Tables

**Table 1:** Biochemistry and haematology results.

Parameters (SI units)	Day of presentation	Day 3 after presentation	Unit	
Haemoglobin: Hb	14.5	12.7	g/dL^1^	(13.0–18.0)
White cell count: WCC	6.7 × 10^9^	5.5 × 10^9^	cells/L^2^	(4.0–11.0)
C-reactive protein: CRP	**88**	**123**	mg/L^3^	(0–10)
Alanine transaminase: ALT	**58**	**449**	U/L^¶^	(0–40)
Aspartate transaminase: AST	**64**	**498**	U/L	(0–35)
Gamma glutamyl transferase: GGT	**60**	**291**	U/L	(0–40)
Alkaline phosphate: ALP	66	**148**	U/L	(35–110)
Bilirubin	8	9	*µ*mol/L	(0–15)
Lipase	48	—	U/L	(20–60)

^1^g/dL = grams/deciliter.

^2^ells/L = cells/Litre.

^3^mg/L = milligrams/Litre.

^¶^U/L = units/Litre.

**Table 2:** Clinical characteristics and outcomes of reported cases of typhoidal acute acalculous cholecystitis.

Reference	Age/sex	Presenting symptoms/signs	Therapy	Complications	Outcome
Lothrop [7]	28 M	Fever, headache, abdominal pain, and generalised guarding	Cholecystectomy antibiotics (unspecified)	Gallbladder perforation	Survived

Garg et al. [8]	35 M	Fever, abdominal pain, and generalised guarding	Cholecystectomy antibiotics (unspecified)	Gallbladder perforation	Survived

Avalos et al. [9]	30 F	Fever, vomiting, headache, abdominal pain, and localised guarding	Ampicillin	Nil	Survived

Lai et al. [10]	36 F	Fever, diarrhea, epigastric pain, localised guarding, and jaundice	Cholecystectomy ceftriaxone	Nil	Survived

Khan et al. [11]	31 M	Fever, diarrhoea, abdominal pain, localised guarding, and jaundice	Ceftriaxone	Nil	Survived

Inian et al. [2]	21 F	Fever, vomiting, diarrhoea, abdominal pain, and guarding	Ciprofloxacin, cefotaxime, and metronidazole	Nil	Survived

Our Case	23 F	Fever, nausea and vomiting, epigastric pain, malaise, and headache	Ceftriaxone, azithromycin, metronidazole, and cholecystectomy	Chest infection	Survived

**Table 3:** Imaging criteria for diagnosis of acute acalculous cholecystitis.

Ultrasound	
*Either two major criteria or one major criterion and two minor criteria *	
Major criteria	
Gallbladder wall thickening > 3 mm	
Striated gallbladder (gallbladder wall edema)	
Sonographic Murphy sign (unreliable if patient is obtunded or sedated)	
Pericholecystic fluid (absent in ascites or hypoalbuminaemia)	
Mucosal sloughing	
Intramural gas	
Minor criteria	
Gallbladder distension (>5 cm in transverse diameter)	
Echogenic bile (sludge)	

Computer tomography	

*Either two major criteria or one major criterion and two minor criteria *	
Major criteria	
Gallbladder wall thickening > 3 cm	
Subserosal halo sign (intramural lucency caused by edema)	
Pericholecystic infiltration of fat	
Pericholecystic fluid (absent in ascites or hypoalbuminaemia)	
Mucosal sloughing	
Intramural gas	
Minor criteria	
Gallbladder distension (>5 cm in transverse diameter)	
High attenuation bile (sludge)	
